# Toxic Metals in a Paddy Field System: A Review

**DOI:** 10.3390/toxics10050249

**Published:** 2022-05-16

**Authors:** Yuanliang Duan, Qiang Li, Lu Zhang, Zhipeng Huang, Zhongmeng Zhao, Han Zhao, Jun Du, Jian Zhou

**Affiliations:** Fisheries Institute, Sichuan Academy of Agricultural Sciences, Chengdu 611731, China; duan9104@126.com (Y.D.); liq7920@126.com (Q.L.); zhanglu425@163.com (L.Z.); h3392078@163.com (Z.H.); 18227552594@163.com (Z.Z.); zhaohan232323@163.com (H.Z.); dujun9100@126.com (J.D.)

**Keywords:** toxic metals, health risk, paddy field, bioremediation, rice–fish co-culture system

## Abstract

The threat of toxic metals to food security and human health has become a high-priority issue in recent decades. As the world’s main food crop source, the safe cultivation of rice has been the focus of much research, particularly the restoration of toxic metals in paddy fields. Therefore, in this paper, we focus on the effects of toxic metals on rice, as well as the removal or repair methods of toxic metals in paddy fields. We also provide a detailed discussion of the sources and monitoring methods of toxic metals pollution, the current toxic metal removal, and remediation methods in paddy fields. Finally, several important research issues related to toxic metals in paddy field systems are proposed for future work. The review has an important guiding role for the future of heavy metal remediation in paddy fields, safe production of rice, green ecological fish culture, and human food security and health.

## 1. Introduction

Metals are typically classified as non-essential and essential metals. Non-essential metals (mercury (Hg), cadmium (Cd), lead (Pb), etc.) have no proven biological purpose, and their toxicity is a function of their concentration. In contrast, essential metals (iron (Fe), zinc (Zn), copper (Cu), etc.) have known biological effects, and toxicity occurs when the metal is metabolically deficient or at a high concentration [1]. In general, any metal or metalloid that is not used in the basic metabolism or is not biodegradable is considered a heavy metal [2]. Heavy metals are defined as those elements with a density greater than 5 g/cm^3^ and an atomic number greater than 20, and include 53 metals [2] (gold (Au), silver (Ag), Cu, etc.). Despite being a metalloid element, arsenic (As) is listed as a heavy metal due to its multiple properties, similar to heavy metals. Heavy metals can be divided into toxic (e.g., Cd), precious (e.g., Au), and radionuclide (e.g., Uranium, U) metals [3]. The term “heavy metal” is considered meaningless at worst and imprecise at best [4,5]; this paper replaces it with “toxic metal” in later descriptions. When the intake of toxic metals exceeds a certain amount, it will do serious harm to humans’ kidneys, nervous system, and fertility, and even cause cancer and death [6,7,8]. In China, Hg, Cd, Pb, chromium (Cr), and As are commonly known as the “five poisons” [9]. Numerous toxic metals (TM) (Cd, Pb, Hg, As, etc.) have been identified as non-essential elements for bodily functions [10] and have been included in the top 20 list of hazardous substances [11]. Therefore, the toxicity, and maximum allowable value of TMs in food and other related issues has been the focus of much attention.

Rice is an extremely important food crop, not only in China but also across the world, with a global cultivation area reaching 145 million hectares. The principle rice-producing areas are distributed in East, South, and Southeast Asia, followed by Mediterranean coastal countries, the United States and Brazil [12]. At present, more than half of the world’s population relies on rice for their calory intake [13], and thus a decline in rice yield will have a significant global impact. By 2050, the growth in population and acceleration of urbanization will require each rice-producing hectare to feed at least 43 people, compared to the current value of 27 people per hectare [14]. Kaur et al. [15] predicted that rice production would need to increase by at least 40% by 2050 to ensure food security. Rice quality is particularly important due to the key role rice plays in the global diet. High-quality rice produced by artificial breeding has been observed to have therapeutic and preventive effects on several human diseases [16]. In addition to nutrients, the phytochemicals present in rice can play a biological role, with antioxidant, anticancer, anti-diabetic, and anti-inflammatory effects [17]. However, poor-quality rice can have a negative impact on human health, causing dizziness, thoracic stuffiness, nausea, vomiting, abdominal discomfort, etc. [18]. Therefore, ensuring the high quality and yield of rice is of great significance.

In 2020, a total of 720–811 million people worldwide were facing hunger, and nearly one-third of the global population (2.37 billion) were not able to get enough food [19]. The yield and quality of rice have been the subject of much interest. TM pollution not only reduces the quality and yield of rice, but also endangers human health and even causes death. The ecological cultivation of aquatic animals and plants has recently attracted the attention of scholars and governments, with its vigorous promotion and active application in practice by farmers. This has consequently improved the quality and yield of agricultural products as well as their taste and safety. An in-depth understanding of the relationships between TMs and rice, and their corresponding removal methods are important prerequisites for effectively improving the food safety of rice. To achieve this goal, in the current paper, we first introduce the effects of TMs on rice. Then, the sources, monitoring methods and measuring instruments of TM pollution, as well as the measures employed to remove TMs and reduce their bioavailability in paddy fields, are discussed in detail. Finally, the research trends and challenges of TM removal or remediation in paddy fields are introduced.

## 2. Effects of TMs on Rice

**Figure 1:**
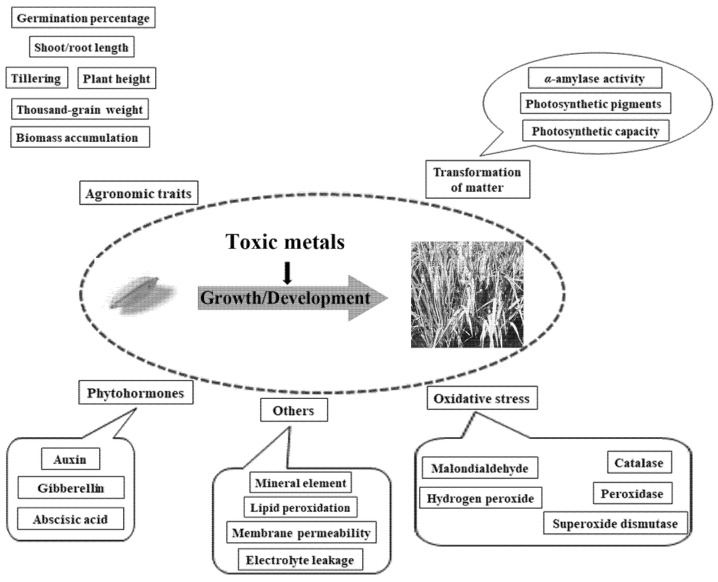
The relationships between toxic metals and the growth, development, metabolism, and nutrient composition of rice.

The relationships between TMs and the growth, development, metabolism, and nutrient composition of rice have been the subject of extensive research (Figure 1). As trace elements, the presence of TMs can have both positive and negative effects on organisms. Low TM concentrations can promote the metabolic activities of organisms and vice versa for high concentrations [20]. TM stress has been observed to have a significant effect on agronomic rice traits, including panicle number per plant, filled grain per panicle, 1000-grain weight, and grain yield per plant [21,22,23,24,25]. In addition, TM stress exerts a great impact on the molecular and gene level of rice, inducing changes of a higher complexity compared to those of the agronomic traits, which are mainly related to the physiological metabolism, variations in enzymes, and the regulation of gene expression [26,27]. Such examples are internal factors that affect the growth and development of rice. Thus, increasing our understanding of the TM mechanisms affecting rice is essential for the prevention and control of TMs.

### 2.1. Effects of TMs on Apparent Indexes and Body Composition of Rice

Highly available Zn concentrations in the soil not only result in rice chlorosis and inhibit plant growth but also reduce Fe concentrations in rice plant shoots to the level considered deficient [28]. Moreover, Cd can prevent the transformation of starch, affect seed germination by inhibiting amylase activity [29], and reduce the chlorophyll content of rice, thus affecting photosynthesis [23,30]. Hg can inhibit germination and seedling growth by damaging the embryo, while Pb destroys endosperm starch solubilization by inhibiting α-amylase activity, and obstructs seed germination and seedling growth [31]. Ni can affect the H-ATpase activity and lipid composition of rice cell membranes [32] and reduce the content of ions (Na, K, and Ca), photosynthetic pigments (chlorophyll and carotenoids), total protein, and organic nitrogen in seedlings [33]. The down-regulation of key metabolic enzymes with excessive Cu, such as α-amylase or enolase, not only affects the starvation absorption of water by seeds, but also leads to the failure of the reserve mobilization process [26]. Lanthanum (La) shoot contents exceeding the toxicity value result in a decline in plant growth and chlorophyll a/b, while peroxidase activity, cell membrane permeability, and proline content in the leaves increase [34,35,36,37]. Tables S1 and S2 report more effects of TMs on the apparent indexes and body composition of rice [38,39,40,41,42,43,44,45,46,47,48,49,50,51,52].

### 2.2. Effects of TMs on the Gene Expression of Rice

Genetic diversity is an important intrinsic factor determining the TM content in rice grains. The TM uptake of different cultivars is varied; for example, Cd-tolerant rice has a certain tolerance to Cd stress [53]. Metal exposure can result in the up- or downregulation of some genes/miRNAs in rice [54] and can consequently alter the corresponding physiological, biochemical, and metabolic properties. Furthermore, TMs can induce the expression of genes involved in numerous biological processes in rice [26,55,56,57], such as signal transduction, ion transport and binding, stress responses, metabolism, etc. However, the expression of genes can also affect the impact of TMs on rice (Table 1). The interaction between genes and TMs is highly complex, and more research is required to clarify the underlying mechanisms.

### 2.3. Effects of TMs with Types and Forms on Rice

The impacts of TMs on rice vary according to the types and forms of TMs, while the same TM can also have different effects on rice. When the concentration of TMs reaches a certain level, it can completely inhibit the germination and growth of rice. For example, high levels of Hg and Pb can inhibit rice germination and seedling growth [21,22] and different types of As can have similar effects [49,70]. The roots of rice are more sensitive to TMs compared to shoots [70]. The regularity effect of TMs on an enzyme is a function of the TM type. For example, Pb, Hg, Cd, and Zn can decrease the superoxide dismutase activity of rice [23,43,45,46], while the opposite has been reported for Cd and Pb [44,47]. Peroxidase is more sensitive to Pb and Cd stress than superoxide dismutase [71]. See Tables S1 and S2 for more information. Therefore, the relationship between TMs and the physiological and biochemical indexes of rice is highly complex and requires verification.

## 3. TM Sources in Paddy Fields

TMs originate from both natural and man-made sources. Natural sources include parent rock weathering [72], volcanic activity [73], and atmospheric deposition [74], etc. Anthropogenic sources refer to pollutants discharged into the environment via human activities, such as industrial emissions [75], pesticides, and chemical fertilizer residues [76], domestic wastewater [77], etc. Anthropogenic activity is the principal source of TMs in paddy fields. In particular, the concentration of TMs in industrialized areas is generally higher than that in non-industrialized areas [78]. The diffusion of TMs from the source to the surrounding areas typically depends on pollution sources, the geochemical status [79], and the surface runoff [80]. Moreover, TMs in the atmospheric phase are transferred to paddy fields via atmospheric deposition. In particular, wet atmospheric deposition of TMs is positively correlated with rainfall depth, while dry deposition is positively correlated with the number of dry days [81]. Sediments are a secondary pollution source of TMs, greatly threatening the safety of aquatic ecosystems. The accumulation or release of TMs in sediments to water bodies depends on the physical, chemical, and biological conditions of the sediment-water interface, such as changes in salinity and pH, biological disturbances, tidal currents, and floods [82].

## 4. TM Monitoring Methods

In view of the importance of food security and the crucial role of rice products in the human diet, TM monitoring and corresponding risk assessments are particularly important. Numerous methods have been developed to investigate the effects of TM pollution in rice paddy fields. Currently, the most popular methods are those using models based on advanced science and technology, for example, the back propagation neural-network [83], time-spectrum feature space [84], and World Food Study (WOFOST) models [85]. Brus et al. [86] employed a multiple linear model to successfully predict the content of TM s in rice grains. The generalized dynamic fuzzy neural network model combines spectral indices with environmental parameters to predict TM concentrations (Cu and Cd), outperforming adaptive-network-based fuzzy interference systems, back-propagation (BP) neural network models, and regression models [87]. Furthermore, the field-scale TM assessment model improves the monitoring of TM stress from its predecessor, the generalized dynamic fuzzy neural network model [88]. Compared with traditional methods (toxicity characteristic leaching, diethylenetriaminepentaacetic acid extraction, and HCl extraction), field capacity-derived soil solution extraction can successfully predict the total Cd content of rice from the tillering to mature stages [89]. The development of science and technology has permitted the gradual application of remote sensing technology to the TM monitoring of crops, for example, the collection of biochemical and hyperspectral data. The coupling of these two data types can be adopted for TM monitoring, indicating the relationship between the TM content in the soil and the cell structure and chlorophyll content in rice canopies or leaves [90,91]. Optical remote sensing typically monitors the internal structure, color, and additional characteristics of crop cells, while microwave techniques focus on the geometric characteristics and morphology of cells. Combining these two technologies can build a robust monitoring model for TM stress in rice, and can also be used to investigate a variety of environmental stresses [92]. The WOFOST model is the most suitable model for the application of remote sensing technology in this field. In particular, integrating remote sensing technology and statistical methods with the world food research model can greatly improve the accurate monitoring of crop TM stress.

## 5. TM Measuring Instruments

For TMs, most exist in nature at natural concentrations, which are relatively low and difficult to detect. However, TMs will be enriched into the human body via food, soil, water, air, and other means, resulting in the destruction of normal human physiological metabolism and a serious impact on human health. Therefore, in order to clarify the TM pollution existing in the current living and ecological environment, it is necessary to do a good job of TM detection with the help of various analytical instruments.

At present, the instruments for the determination of TMs mainly include atomic absorption spectrometer, inductively coupled plasma spectrometer, atomic fluorescence spectrometer, inductively coupled plasma mass spectrometer, voltammetric analyzer, etc. Because of its sensitivity, accuracy, and simplicity, atomic absorption spectrometers have been widely used in the analysis of TMs in agriculture, food, and environmental monitoring [93,94,95], etc. An inductively coupled plasma spectrometer can detect multiple elements (metal elements and non-metallic elements) in the sample [96,97], and its sensitivity is relatively high. Compared with atomic absorption spectrometry, an inductively coupled plasma spectrometer is suitable for the determination of more than three samples at the same time. Atomic fluorescence spectrometers have the advantages of low price, low detection cost, low detection limit and high sensitivity [98,99,100]. It is easier to popularize than other detection instruments. Inductively coupled plasma mass spectrometry has good detection limit, scanning ability, and relatively high sensitivity; moreover, it can determine multiple elements at the same time and can determine and identify isotopes [101,102]. However, compared with other analytical instruments, the detection cost of inductively coupled plasma mass spectrometers is relatively high, and the detection limit of some elements is limited. A voltammetric analyzer can have a better detection effect for the monitoring of trace metals [103,104]. It can have a certain sensitivity and precision for the determination of TMs in some substances by means of dissolution analysis, so the accuracy of the final monitoring results is relatively high. It is relatively simple to use and is an important analytical instrument in trace analysis.

## 6. Remediation of TMs from Paddy Fields

Reducing TM content in rice has been the focus of much research, typically via enhancing the stability of pollutants in the soil, reducing soil surface pollutant concentrations, modifying the ability of rice to absorb and transport TMs, and so on. Studies on reducing plant available TMs in the environment generally concentrate on soil remediation techniques: turnover and dilution, in situ stabilization via chemical improvers, and bioremediation [105].

### 6.1. Remediation of TMs with Soil Amendments

#### 6.1.1. Metal Soil Amendments

At present, the majority of relevant research is directed toward soil amendments due to their rapid and efficient effects. In particular, soil amendments are a class of compounds containing Ca, Fe, etc. TM adsorption by soil amendments occurs via physical adsorption, surface complexation, and ion exchange [106], which convert TMs to non-biousable forms [107]. The bioavailability of TMs in soil is the result of the interaction of organic matter, ions, redox conditions, and soil pH [108]. Soil pH exerts a great influence on TM content in rice; for example, the optimal pH for the adsorption of Ni (II) and Cu (II) is 6 and 5, respectively [109]. pH levels in paddy fields can be increased by applying liming and red mud [106,110]. As well as impacting pH levels, red mud also improves the microbial composition in paddy fields and increases the activity of urease, acid phosphatase, and catalase in the soil [110]. Fe exhibits high bioavailability and does not exert adverse effects on rice quality and yield [111]. Adding Fe to paddy fields can reduce the absorption of TMs by rice and increase the elemental contents of Fe, Cu, Mn in rice grains, and Zn in rice plants [112]. Moreover, the application of Fe-containing materials can effectively reduce the concentration of As in soil solutions and rice grains, with zero-valent Fe demonstrated to be particularly powerful. Makino et al. [113] attributed this to the formation of arsenic sulfide. Moreover, Yu et al. [114] determined a significant positive correlation between As and Fe, suggesting that Fe-containing amendments may have an indirect influence on the fractionation of soil As and biological effects to ease the As for rice. The application of metals or additional complexes can enhance the amount of iron plaque, which is composed of crystallized and amorphous iron oxides, hydroxides, etc. [115], on the root surface [116]. This technique can also enhance the interception of TMs by rice roots.

#### 6.1.2. Non-Metallic Soil Amendments

Non-metallic soil amendments are mainly compounds containing silicon (Si), organic matter, etc. Si soil amendments have been the focus of much research due to their ability to actively induce the molecular expression of Cd tolerance in rice leaves. The addition of Si to paddy fields can reduce the As and Cd content in rice, alleviate abiotic stress, and increase rice yields, while also significantly improving the uptake of N, P, and K by rice [117]. Immobilized metals are typically bound in soil organic matter components and exist as an organic binding state [118], thus facilitating research on organic soil amendments. Common biochar contains straw, hull, etc. [119,120]. Biochar can effectively fix TMs and reduce their bioavailability and mobility in soil [118]. Moreover, biochar can directly or indirectly affect indigenous microorganisms by changing the physical and chemical properties and TM content of sediments [121]. However, biochar has also been reported to induce oxidative stress in rice [122], and thus any potential negative effects of biochar must be considered in agricultural and environmental applications. Furthermore, the application of non-metallic elements (e.g., Se and S) can alleviate the toxic effects of TMs on rice [116,123].

#### 6.1.3. Nanoscale Soil Amendments

Nanoscale soil amendments have recently been the focus of much research, achieving promising results. For example, Nano-Si has a positive impact on the yield and growth of rice in polymetallic contaminated soil and can reduce TM content in grains [124]. Moreover, CuO nanoparticles have been reported to accelerate the arrival of the rice heading stage, shorten the plant life cycle, and reduce As accumulation in grains [125]. Biochar nanoparticles have a high adsorption affinity for Cd, thus reducing the toxicity of Cd in rice. This is particularly true for biochar nanoparticles prepared under high temperature conditions, manifested as increased biomass, root activity, and chlorophyll content in rice plants [122]. However, the toxicity and outcome of co-existing metals with nanoparticles remain unclear. The negative impact exerted by CuO nanoparticles on plant growth is more significant than that of bulk particles [126]. High ZnO nanoparticle concentrations are able to enhance the content of bioavailable Cd in rhizosphere soil. The addition of ZnO nanoparticles at high concentrations to soil containing low levels of Cd can significantly promote Cd accumulation in rice [127].

#### 6.1.4. Composite Soil Amendments

Multiple TMs are typically present in paddy fields; thus, applying composite soil amendments rather than a single component is required. The Cd bioavailability in the rhizosphere of rice can decrease by 92–100% from the tillering stage to maturity via the application of Ca-Si-rich composite minerals. In addition, Si deposition on the rice root cross-section has been observed to significantly increase following the application of a Ca-Si-rich composite mineral treatment, which consequently enhances the storage of Cd in roots and reduces the translocation of Cd from the root to the shoot [128]. Sulfur and iron-modified biochar amendments can significantly increase the amount of iron plaque on the root surface, facilitating the transition of Cd to binding states (such as Fe-Mn oxide) and reducing Cd concentration in contaminated soil and rice grains [116]. The combined application of biochar and lime reduces Pb availability in soil and Pb accumulation in brown rice at a greater rate compared to the corresponding single applications [129]. Furthermore, integrating ferric oxide and calcium sulfate into a single amendment can effectively reduce the bioavailability of Pb and Cd in soil and the content of Cd, As, and Pb in rice grains [130]. Moreover, Honma et al. [131] demonstrated the ability of prolonged flooding with short-range-order iron hydroxide and rainfed management combined with converter furnace slag to reduce both the Cd and As uptake of rice.

### 6.2. Bioremediation of TMs

Bioremediation is a low-cost technique that has a limited impact on the environment, and includes phytoremediation, microbial remediation, animal remediation, etc. Crop rotation and intercropping are common TM remediation methods that can effectively ensure the safety and yield of rice and restore metal-contaminated soil [107,132,133], with examples including wheat-rice rotation, oilseed rape-rice rotation, rice-water spinach intercropping, etc. The composition and sources of environmental microbiota play a key role in the health and productivity management of sustainable agriculture. The application of microorganisms to paddy fields contaminated with TMs is a newly developed remediation method. Scholars have identified a reduction in TMs with resistant bacteria in rice grains and have highlighted the potential of bioremediation for contaminated soil. For example, Cd transporters (OsHMA2 and OsNramp5) in rice roots can experience down-regulation following inoculation with *Stenotrophomonas maltophilia*. This may be an internal factor affecting Cd content in rice [134]. Lin et al. [135] determined *Stenotrophomonas acidaminiphila*, *Pseudomonas aeruginosa*, and *Delftia tsuruhatensis* to be Cd tolerant, effectively reducing the enrichment of Cd in rice grains. In particular, *P. aeruginosa* is considered to be a multi-metal-resistant bacterium. Animal remediation technology refers to the absorption, transfer, or degradation of TMs through the food chain of soil animals, and research in this field is relatively limited. The backbone of animal remediation is microbial remediation [136]. For example, earthworms have the ability to alter the structure and permeability of soil, and form the basis of the most commonly used remediation method for TM-contaminated soil.

### 6.3. Field Management

Water management approaches are easy to operate and are commonly adopted. For example, continuous culture flooding is an effective method for reducing TM content in rice grains [137], yet it has an increased risk of As accumulation [113]. In addition, the aerobic conditions created by the release of water [138] in aerobic treatments can increase Cd concentrations [139]. Flooding in paddy fields may cause sulfide mineral precipitation, significantly reducing trace metal solubility [140], as well as the affinity for metals in the rhizosphere and iron plaque on the root surface [137]. Although the drying-wetting cycles of soil promote the release of metals into the water, Honma et al. [141] determined that intermittent irrigation (3-day flooding and 5-day no-flooding) can simultaneously reduce the accumulation of As and Cd in grains. Current research on the combined benefits of intermittent and aerobic irrigation demonstrates the ability of intermittent irrigation to reduce the Cd content in grains and increase rice yields [142].

### 6.4. Planting Methods and Varieties

Furthermore, the cultivation, season, and variety of rice may affect the relationship between rice and TMs during the production process. Deng et al. [143] demonstrated greater Cd and Pb contents in brown rice, straw, and roots via the direct seeding method compared with manual transplanting and seedling throwing. Farooq and Zhu [144] and Yi et al. [145] determined that early and late planting of rice impacted the Cd content in white rice. Differences in rice varieties are attributed to gene differences. Under low and moderate soil Cd pollution, japonica rice cultivars are more suitable than indica rice [146]. Dry season varieties are more tolerant to arsenite or arsenate than rainy season varieties [147]. Studies have also reduced the toxicity of TMs to rice by inserting or removing certain genes via genetic engineering and cross breeding [57,61,65,68]. For example, low Cd accumulation may reduce Cd absorption by inhibiting the bioavailability of Cd in the rhizosphere or by decreasing Cd transport [148]. Thus, the genotype, environment, and their interaction are considered the most significant factors affecting the TM content in rice grains.

## 7. Recent Trends and Challenges

The importance of rice to human beings and TMs may cause great harm to human beings through bioaccumulation, which will lead to research on TMs in rice fields becoming a hot issue. The relevant research on the treatment of TM pollution in paddy fields around the world mainly includes: (1) changing the existing state of TMs in soil, making them fixed or stable, and then reducing the activity of TMs; (2) changing the planting system to reduce the absorption of TMs by rice; (3) extracting TMs from rice fields, so as to reduce the concentration of TMs in rice fields. Since 2011, the number of papers published in the field of TM remediation in paddy fields has increased rapidly. These studies mainly focused on five TMs: Cd, Pb, As, Cu, and Zn, which indirectly showed that rice fields around the world were mainly polluted by these heavy metals. In addition, there was also a small amount of research on rare earth metals. Among these research hotspots, the remediation and treatment of Cd pollution in paddy fields was the main, and the passivation remediation fixation/stabilization technology was the most. However, the best effect of reducing Cd was the planting of low-accumulation Cd varieties, which could reach 74.50% [149].

It is very difficult to completely remove TMs from rice fields. At present, scholars mainly focus on passivating TMs, reducing their activity, and reducing their absorption by rice. In addition, at present, most of the treatment technologies of TM pollution in rice fields are still in the stage of laboratory or field experiment and demonstration, and there are few real engineering applications, so they have great market potential in the future. In order to realize the simultaneous production and repair activities, we can develop environment-friendly, long-lasting, and high-efficiency passivators for moderate and mild TM pollution; for severe pollution, the combination of soil elution technology and passivation remediation technology or phytoremediation technology can be used; for microbial remediation, especially the screening and cultivation of microbial strains, further research is needed. Due to the differences in soil properties, there are few universal technologies that can be popularized in a large area. Therefore, it is particularly important to develop efficient and lasting comprehensive prevention and control technology for toxic metal pollution in paddy fields according to local conditions.

## 8. Conclusions

TMs may affect human growth and development, physiological metabolism, etc., and may cause diseases and even death. TMs enter the food chain via organisms located at the bottom of the food chain, and their concentration and toxicity are subsequently amplified as they move further up the food chain. Consuming a certain amount of food contaminated by TMs can threaten an individual’s health. Thus, humans (who are at the top of the food chain) face great health risks, as they risk TM exposure principally through food intake. Rice is more important than fish in terms of the risk of metal exposure in the human diet, and arsenic requires particular attention. Grain crops (e.g., rice) that grow on soil/water polluted by TMs not only experience a reduction in yield and quality but also enrich a large amount of TMs. To reduce the threat of TMs to human health, measures must be taken from the source. In particular, uncontaminated soil and water bodies can guarantee the production of healthy food, which is key to human health. Therefore, the research and exploration of the technical methods of heavy metal removal or remediation in rice fields is of great significance to human food safety and health.

## Figures and Tables

**Table 1:** Toxic metal-related genes.

Gene/Protein	Function	References
*OsHMA1*, *OsHMA2*, *OsHMA3*, *OsHMA4*, *OsHMA5*	involves the transfer of toxic metals and reduces toxic metals concentrations in rice grains	[55,57,58,59]
*OsGSTL2*	provides tolerance for toxic metals and other abiotic stresses	[60]
*OsLEA4*	contributes to toxic metal, drought and salt tolerance in rice plants	[61]
*CAL1*	reduces cytosolic Cd concentration by promoting the secretion of Cd into the extracellular space and chelating Cd in the cytosol	[56]
*MTP11*	Mn tolerance via intracellular Mn compartmentalization	[62]
*OsATX1*	promotes the redistribution of Cu from old leaves to developing tissues and seeds, as well as the root-to-shoot Cu translocation in rice	[63]
*OsMTs*	promotes rice multiple stress tolerance	[64]
*WaarsM*	induces As volatilization and methylation, thus reducing As content in grains	[65]
*OsMRLK*	promotes rice development and multiple stress tolerance	[66]
*OsLCT1*, *OsHMA2*, *OsZIP3*	alleviates the oxidative stress of Cd and Zn, decrease the translocation and accumulation of Cd to grains	[57]
*OsVMT*	enhances the content of Zn and Fe in polished rice	[67]
*OsNRAMP1*, *OsNRAMP5*	contributes to the uptake of Cd and Mn in rice	[68,69]

## Data Availability

All available data is included in the paper.

## Supplementary Materials

The following supporting information can be downloaded at: https://www.mdpi.com/article/10.3390/toxics10050249/s1, Table S1: Effects of toxic metals on the apparent indexes and body composition of rice (Promote/Increase). Table S2: Effects of toxic metals on the apparent indexes and body composition of rice (Inhibit/Reduce).
