# Supplementary material for: Toxic Metals in a Paddy Field System: A Review
Source: Toxics. 2022 May 16;10(5):249. doi: 10.3390/toxics10050249 (PMC9148070; doi:10.3390/toxics10050249)
Supplement: Supplementary file 1 [file toxics-10-00249-s001.zip › Toxics-SM-template-updated - Copy.pdf]

toxics MDPI

Supplementary Materials: Toxic Metals in a Paddy Field System: A Review

Yuanliang Duan, Qiang Li, Lu Zhang, Zhipeng Huang, Zhongmeng Zhao, Han Zhao, Jun Du and Jian Zhou

Table S1. Effects of toxic metals on the apparent indexes and body composition of rice (Promote/Increase)

Organization/Substance/ Parameters	Influence of toxic metals		Toxic metal species	References
	Concentrations (ppm)	Degree		
Germination percentage, shoot/root length, etc.	Sb [III, 0–50]; La [0–6.946]	Shoot length (~18.7%); root length increase	$La(NO_3)_3$	[36,38]
Transformation ratio of dry matter	Sb [V , 5–50]	The transformation ratio of dry matter exhibited a slight increase	na	[38]
α–amylase activity	Sb (V) [5–50]	Sb (V) can increase the activity of α–amylase	na	[23,38]
Plant height	Cd [0–0.0112]; La [0–30]	Plant height increased slightly	$CdCl_2$; $LaCl_3$	[34]
Tillering ability	As [0–1]	This effect reached a significant level (~12.60%)	$Na_2HAsO_4{\cdot}7H_2O$	[39]
Thousand–grain weight	na	na	na	na
Biomass accumulation	Cu [0.00065–0.065]	The biomass of rice increased significantly at low (<0.065 mg L^{-1}) Cu (~30.99%)	$CuSO_4$	[40]
Net photosynthesis and photosynthetic capacity	As [0–0.2]	Net photosynthesis (Pn) and photosynthetic capacity were increased slightly	Dimethylarsenic acid (DMAA)	[41]
Photosynthetic pigments	na	na	na	na
Gibberellin	na	na	na	na
Abscisic acid	Pb [1000]	Abscisic acid was significantly increased	$Pb(NO_3)_2$	[42]
MDA	Pb [2.072–1200], Hg [2.006], Cd [0–100], Zn [200], La [69.455–208.365]	Cd treatment had a substantial influence in MDA (~177.99%)	$PbCl_2$, $Pb(NO_3)_2$, $HgCl_2$, $CdCl_2$, $CdCl_2{\cdot}5H_2O$, $CdCl_2{\cdot}2.5H_2O$, $ZnSO_4{\cdot}7H_2O$, $La(NO_3)_3$	[23,27,37,43–46]
POD	La [0–900], Pb [400–1200], Cd [50], Zn [200], La [0–13.891]	Significant changes were observed in POD (~104.89%)	$LaCl_3$, $Pb(NO_3)_2$, $CdCl_2{\cdot}5H_2O$, $ZnSO_4{\cdot}7H_2O$, $La(NO_3)_3$	[34,37,44,45]
SOD	Pb [400–1200]; Cd [150]	Significant changes were observed in SOD (~230.13%)	$Pb(NO_3)_2$, $CdCl_2{\cdot}2.5H_2O$	[44,47]
CAT	Pb [2.072–1200], Cd [50–150], Zn [200], La [0–13.891]	Significant changes were observed in CAT (~86.49%)	$PbCl_2$, $Pb(NO_3)_2$, $CdCl_2{\cdot}2.5H_2O$, $CdCl_2{\cdot}5H_2O$, $ZnSO_4{\cdot}7H_2O$, $La(NO_3)_3$	[37,43–45,47]
H_2O_2	Pb [400–1200], Cd [50–150], Hg [2.006], La [69.455–208.365]	The content of H_2O_2 increased significantly (~156.01%)	$Pb(NO_3)_2$, $CdCl_2{\cdot}2.5H_2O$, $HgCl_2$, $La(NO_3)_3$	[27,37,43,44,46,47]
Others	La [0–900], Pb [2.072–1200], Hg [2.006], Cd [50–100], Zn [200]	Deterioration of membranes, membrane permeability increase, leakage of electrolytes, etc.	$LaCl_3$, $PbCl_2$, $Pb(NO_3)_2$, $HgCl_2$, $CdCl_2{\cdot}5H_2O$, $CdCl_2{\cdot}2.5H_2O$, $ZnSO_4{\cdot}7H_2O$	[27,34,43–46]

Note: The Sb, MDA, POD, SOD and CAT represent antimony, malondialdehyde, peroxidase, superoxide dismutase and catalase, respectively. The "na" represents nothing or not clear. For the convenience of comparison and analysis, the units are unified as ppm.

Table S2. Effects of toxic metals on the apparent indexes and body composition of rice (Inhibit/Reduce).

Organization/Substance/ Parameters	Influence of toxic metals		Toxic metal species	References
	Concentrations (ppm)	Degree		
Germination percentage, shoot/root length, etc.	Sb [III, 0.001–1], Sb [Ⅴ, 0.001–1], Pb [2.072–1000], Hg [2.006–20.06], Cd [0.1124 – 28.10], Cu [12.70 – 127.00], Zn [16.35], Ni [0–1000], La [138.91–208.365], As [Ⅴ, 0–20], As [III, 0–20]	Germinating seeds was greatly inhibited, even no root and shoot formation was observed in a high concentration.	$PbCl_2$, $Pb(NO_3)_2$, $HgCl_2$, $CdCl_2$, $Cd(NO_3)_2$, $CuSO_4$, $ZnSO_4$, $NiCl_2$, $La(NO_3)_3$, $Na_2HAs_3O_4{\cdot}7H_2O$, As_2O_3	[21,22,24,26,29,30,31,33,36,38,42,48,49]
Transformation ratio of dry matter	Sb [III, 0.001–1], Sb [Ⅴ, 0.2–1]	The transformation ratio of dry matter gradually decreased from 25.7% to 46.05%	na	[38]
α–amylase activity	Sb [III, 0.001–1], Sb [Ⅴ, 0.1–1], Pb [20.72–1000], Hg [2.006], Cd [0.1124 – 22.48], Cu [12.70–127.00]	α–amylase activity in germinating seeds is significantly decreased (~85.2%)	$PbCl_2$, $Pb(NO_3)_2$, $HgCl_2$, $CdCl_2$, $CuSO_4$	[24,26,29,31,38,42]
Plant height	La [150–900], Pb [0–248.64], Cd [0.1124–35], As [0–8]	Plant height decreased significantly with increasing toxic metals concentrations (~49%)	$LaCl_3$, $Pb(NO_3)_2$, $CdCl_2$, $CdSO_4{\cdot}8/3H_2O$, $Na_2HAsO_4{\cdot}7H_2O$,	[23,25,34,39,50,51]
Tillering ability	Pb [0–1200], Cd [30–150], As [2–8]	Tillers occurred slowly and tiller number decreased significantly (~55%)	Pb $(NO_3)_2$, $CdCl_2{\cdot}2.5H_2O$, $Na_2HAsO_4{\cdot}7H_2O$	[25,27,39,44,47,50]
Thousand–grain weight	Pb [400–1200], Cd [30], As [0–8]	Toxic metals significantly reduced 1000–grain weight of rice (~17.94%)	$Pb(NO_3)_2$, $Na_2HAsO_4{\cdot}7H_2O$	[27,39,44,50]
Biomass accumulation	Pb [0–248.64], Hg [2.006–20.06], Cd [150], Cu [0.065–6.5], As [0–8]	The biomass of rice decreased significantly (~62%), and the reduction in dry weight observed in shoot, root, and leaf	$PbCl_2$, $Pb(NO_3)_2$, $HgCl_2$, $CdCl_2{\cdot}2.5H_2O$, $CuSO_4$, $Na_2HAsO_4{\cdot}7H_2O$, Dimethylarsenic acid	[21,22,25,39,40,41,47]
Net photosynthesis and photosynthetic capacity	Cd [1.124–150], Ni [5.87–29.35], As [0.8–1.6]	Net photosynthesis and photosynthetic capacity were decreased significantly	$CdCl_2$, $CdCl_2{\cdot}2.5H_2O$, $CdSO_4{\cdot}8/3H_2O$, $NiCl_2$, Dimethylarsenic acid	[41,47,51,52]
Photosynthetic pigments	Pb [400–1200], Cd [0.1124–100], Zn [200], Ni [0–1000], As [Ⅴ, 0–20], As [III, 0–20]	Pb stress inhibited photosynthetic pigments significantly, i.e., Chl a (~79.65%), Chl b (~85.21%), total Chlorophyll contents (Chl a + Chl b) (~80.98) and carotenoids (~87.76%)	$Pb(NO_3)_2$, $CdCl_2$, $CdCl_2{\cdot}5H_2O$, $CdCl_2{\cdot}2.5H_2O$, $CdSO_4{\cdot}8/3H_2O$, $Cd(NO_3)_2$, $ZnSO_4{\cdot}7H_2O$, $NiCl_2$, $Na_2HAs_3O_4{\cdot}7H_2O$, As_2O_3	[23,27,30,33,44 – 46,49,51]
Gibberellin	Pb [1000]	lead promotes GA inactivation and inhibited GA signal transduction in rice seeds	$Pb(NO_3)_2$	[42]
Abscisic acid	na	na	na	na
MDA	na	na	na	na
POD	Cd [50–100]	POD activity showed a decreasing trend (~14.88%)	$CdCl_2{\cdot}2.5H_2O$	[46]
SOD	Pb [2.072], Hg [2.006], Cd [0.01124–100], Zn [200]	SOD activities were decreased significantly (~51.29%)	$PbCl_2$, $HgCl_2$, $CdCl_2$, $CdCl_2{\cdot}2.5H_2O$, $CdCl_2{\cdot}5H_2O$, $ZnSO_4{\cdot}7H_2O$	[23,43,45,46]
CAT	Hg [2.006], Cd [0.01124–100]	CAT activity showed a decreasing trend (~38.93%)	$HgCl_2$, $CdCl_2$, $CdCl_2{\cdot}2.5H_2O$	[23,43,46]
H_2O_2	Pb [2.072]	H_2O_2 content showed a decreasing trend (~27.50%)	$PbCl_2$	[43]

Others	Ni [0–1000]	Nickel greatly reduced the concentrations of Na, K, and Ca in rice	$NiCl_2$	[33]

Note: The Sb, MDA, POD, SOD and CAT represent antimony, malondialdehyde, peroxidase, superoxide dismutase and catalase, respectively. The "na" represents nothing or not clear. For the convenience of comparison and analysis, the units are unified as ppm.
